# Brain derived neutrophic factor (BDNF) coordinates lympho-vascular metastasis through a fibroblast-governed paracrine axis in the tumor microenvironment

**DOI:** 10.14800/ccm.1566

**Published:** 2017-07-10

**Authors:** Tilahun Jiffar, Turker Yilmaz, Junegoo Lee, Yair Miller, Lei Feng, Adel El-Naggar, Michael E. Kupferman

**Affiliations:** 1 Department of Head and Neck Surgery, MD Anderson Cancer Center, Houston TX 77030, USA; 2 Department of Biostatistics, MD Anderson Cancer Center, Houston TX 77030, USA; 3 Department of Pathology, MD Anderson Cancer Center, Houston TX 77030, USA

## Abstract

It has long been known that the tumor microenvironment contributes to the proliferation and survival of neoplasms through the constant interaction with the stromal and immune compartments. In this investigation, we explored the role of cancer-associated fibroblasts (CAFs) in the regulation of the tumor microenvironment in head and neck squamous cell carcinoma (HNSCC) though a complex intercellular BDNF-TrkB signaling system. Our studies show that conditioned media derived from patient-derived CAFs promoted HNSCC cell proliferation, in vitro cell migration, cell invasion and chemotherapy resistance, compared to normal fibroblasts. Furthermore, examination of the *in vivo* impact of CAF pathophysiology in the tumor microenvironment in animal xenograft models revealed that HNSCC cell lines in combination with CAFs promoted tumor growth and increased incidence of lymphovascular metastasis as compared to injection of tumor cells or CAF cells alone. Using pharmacological and genetic alterations, we mechanistically demonstrate the critical importance of BDNF-TrkB signaling in the tumor microenvironment. These investigations further support the rationale for BDNF/TRKB targeted therapy against in the treatment of HNSCC.

## Introduction

The involvement of regional lymph nodes is the primary mode of metastasis for most solid tumors, a clinical phenotype that has a profound impact on therapeutic strategies and survival in cancer patients. For head and neck squamous cell carcinoma (HNSCC), lymphatic metastasis occurs in over 50% of all patients, and its presence portends for the development of distant metastasis and adverse prognosis. Although improvements have been achieved in surgical techniques, radiation therapy and systemic treatments, the overall five-year survival for patients with lymphatic metastasis remains unaltered. While molecular-targeted agents have demonstrated anti-cancer efficacy in pre-clinical models and clinical trials for HNSCC ^[1–5]^, progress in elucidating the fundamental mechanisms of lymphatic metastasis has yielded few promising targets. Although metastasis to the regional nodes represents a coordinated cellular and molecular ballet of distinct cell populations in the tumor microenvironment, the fundamental contributions of these protagonists to lymphatic metastasis are still poorly understood.

The oncogenic milieu is comprised not only of primary tumor cells, but also endothelial and inflammatory cells, that have been shown to promote carcinogenesis, tumor growth and metastasis. Yet, the major cellular component of the stromal architecture is the fibroblastic compartment, which has emerged as a functional contributor to the tumor microenvironment. Termed cancer-associated fibroblasts (CAFs), these mesenchymal cells have been shown to mediate diverse pro-oncogenic processes, including malignant transformation, inflammation, angiogenesis and invasion. Clinically, the extent of fibroblastic response is has been linked to survival outcomes among patients with various cancers, including HNSCC. Although recent evidence implicates CAF-controlled signaling pathways in modulating the tumor microenvironment, the molecular mechanisms underlying these CAF-mediated processes are incompletely understood ^[6–10]^. While the elucidation of some of these micro-environmental cues has shed light on how bi-directional communication between CAF and tumor cells impacts malignant progression, whether CAFs are instrumental in vascular and lymphatic metastasis has not been fully explored to date.

Investigation over the last twenty years has implicated neurotrophin signaling in multiple biological processes ^[11–13]^, and several lines of evidence demonstrate the role of the brain-derived neurotrophic factor (BDNF) and its cognate receptor, TrkB, in various cancers ^[14–19]^. Previous studies have revealed roles for BDNF-TrkB in malignant transformation, tumor progression, metastasis, angiogenesis, and therapy resistance ^[20–27]^. These studies suggest that the BDNF-TrkB axis may be a critical component of multi-step tumor progression in carcinomas, although the precise mechanisms for its contribution are incompletely understood. While in certain disease models, tumor-derived BDNF instigates abnormal intracellular pathway upregulation in an autocrine and paracrine manner, whether CAF-derived BDNF ligand contributes to a putative CAF-tumor synapse in potentiating lymphovascular invasion has not been explored to date.

In this study, we demonstrate the important role of human tumor-derived CAFs in mediating tumor metastasis that is mechanistically driven by the BDNF-TrkB signaling pathway. Specifically, using model systems of squamous cell carcinoma, we show that CAFs directly control the biological behavior of aggressive malignancies *in vitro* and *in vivo*. Further, CAF-derived BDNF contributes mechanistically to the development of a pro-invasive and pro-angiogenic metastatic milieu that promotes aggressive tumor behavior and adverse outcomes among patients with squamous cell carcinoma.

## Methods

### Cell lines and reagents

The head and neck squamous cell carcinoma cell lines OSC19, UMSCC1, Tu138, FADU, UMSCC-22A cells were maintained as described previously ^[28, 29]^. The OSC19-Luc cell line is a stable transfectant constitutively expressing luciferase and GFP ^[30]^. Normal human fibroblasts (Detroit 551) were purchased from ATCC. Recombinant human BDNF and EGF were obtained from Peprotech (Rocky Hill, NJ). The following antibodies were utilized: TrkA (sc-14024, Santa Cruz Biotechnology, Inc.), TrkB (sc-8316), TrkC (sc-117), NGF (sc-548), BDNF (sc-546), vimentin (sc-51719), GAPDH, MAPK (9107, Cell Signaling, Inc.), phospho-MAPK (9101), STAT3 (9132), phospho-STAT3 (9145), AKT (9272), phospho-AKT^ser473^ (3787), Twist (4119), E-cadherin (4065), N-cadherin (4061), β-catenin (9562), PCNA (2586), Slug, Snail and LYVE-1.

### Isolation of cancer-associated fibroblast

Cancer-associated fibroblasts were harvested from human HNSCC tumor tissues by using an explant technique. All procedures were performed under an IRB-approved protocol. Briefly, ex vivo biopsies were taken in a sterile manner from surgically resected tumors within 30 minutes of tumor resection. Tissues were cut with scissors and scalpel into smaller pieces of 2–3 mm and planted on a tissue culture dish in fibroblast growth medium-2 (Lonza) containing 1% penicillin/streptomycin (Invitrogen) at 37°C in a 5% CO_2_-humidified atmosphere. CAFs grew around the explants and were cultured for approximately 2–3 weeks. Pieces of tissue fragments were removed, and cells were trypsinized and seeded at 70% to 90% confluence and cultured in complete medium. When cells reached 95% to 100% confluence (~1.7–2.0 × 10^4^ cells/cm^2^), they were washed with PBS, incubated with serum-free medium for 1 hour, washed again, and incubated for 24 hours in serum-free medium.

### siRNA and plasmid transfections

Small interfering RNA targeting BDNF, GAPDH (Ambion) and AKT (Sigma) were transfected into cells according to the manufacturer’s protocol. Confirmation of target gene downregulation was confirmed after 48 hours. Short-hairpin RNA (shRNA) constructs targeting TrkB or BDNF were purchased from Origene (Rockville) and were introduced into cells via retroviral infection according to the manufacturer’s protocol and selected with puromycin (1 μg/ml).

### Western blotting (WB)

Cells were grown to 80% confluency, washed with PBS and lysed for 30 minutes on ice (Tris-HCL 50mM, NaCl 100 mM, Triton-X 1%, deoxycholute 0.5%, MgCl2 10mM, NaVO3 1mM, NaF 50mM, PMSF 1mM, protease inhibitor in PBS). SDS-PAGE analysis was performed, and membranes were incubated overnight at 4° with antibodies directed against the indicated proteins. Membranes were washed, incubated with the appropriate secondary antibodies and exposed to Lumi-Light Western Blotting Substrate kit (Roche Diagnostics Corporation, Indianapolis, IN). Images were analyzed with *ImagePro* (MediaCybernetics, Bethesda, MD) and *Prism* (GraphPad Software, La Jolla). Densitometry data were analyzed by utilizing either conventional Student’s *t* test or ANOVA followed by *post hoc* comparisons based upon modified Newman-Keuls-Student procedure, where appropriate. Results are reported as mean +/− SEM. A *p* value of < 0.05 was considered significant and all are two-tailed.

### ELISA experiments

Supernatants were collected from treated cells after 24 hours, filtered, centrifuged and stored at −80°. Levels of secreted BDNF were measured in the cultured supernatant with BDNF E_max_® ImmunoAssay System using the manufacturers’ instructions (Promega, Madison, WI). Samples were measured in triplicate, and the mean value was used for analysis. Protein levels were analyzed by the ANOVA test followed by *post hoc* comparisons based upon modified Newman-Keuls-Student procedure. Results are reported as mean +/− SEM. A *p* value of < 0.05 was considered significant and all are two-tailed.

### Measurement of apoptosis

Cells were seeded in 6-well plates at a density of 3×10^5^ and treated the next day with various inhibitors. After 24 hours post-treatment both detached and attached cells were harvested and washed once in PBS. Cells were centrifuged at 200*g* for 5 minutes and the pellet was resuspended in annexin binding buffer and incubated in annexin V for 15 minutes in the dark according to the recommendation of the manufacturer (EMD Chemicals, New Jersey). Propidium iodide flow cytometric analysis were performed on Gallios (Beckman, Coulter, CA).

### Measurement of cell proliferation

The anti-proliferative effects of chemotherapeutic agents on CAFs were determined by a 3-(4,5-dimethylthiazol-2-yl)-2,5-diphenyltetrazolium bromide (MTT) assay, as previously described ^[2]^. Differences between groups were analyzed by utilizing either conventional Student’s *t* test or ANOVA followed by *post hoc* comparisons based upon Bonferroni’s Multiple Comparison Test, where appropriate. Results are reported as mean +/− SEM. A *p* value of < 0.05 was considered significant, and all are two-tailed.

### Measurement of tumor-associated cytokines (TACs)

TACs were measured by multiplex bead assay (36 factors) as previously described ^[31]^. Multiplex bead assays were done with BioSource Multiplex Assays for Luminex (Invitrogen) in a 96-well format according to the BioSource protocol. CAF concentrations were calculated based on a standard curve derived by performing six serial dilutions of a protein standard in assay diluent. Samples were tested in duplicate, and the mean value was used for analysis. If the mean of the duplicate values for all factors in an individual sample varied by >25%, the sample was retested. Individual factors were excluded from analysis if ≥50% of the samples were out of range or extrapolated. When out-of-range values were included in the analysis, the highest value (measured or extrapolated) across all samples was substituted for values above the upper limit of detection. For values below the lower limit of detection, half the lowest value was substituted.

### Human tumor analysis and immunohistochemistry

Fresh-frozen and paraffin-embedded HNSCC tumors were obtained from the MD Anderson Cancer Center Head and Neck Tumor Tissue Repository under an Institutional Review Board approved protocol. The HNSCC tissue array was designed as previously described ^[32]^. Reactivity to TrkB or BDNF was assessed immunohistochemically based on the percentage of positive cells in 10 consecutive high-power fields (1000 cells) and the intensity of staining graded from 0 to 3+ (0=<10% cells with weak stain, 1=10–25% cells with weak to intermediate stain, 2=25–50% cells with intermediate stain, 3=51–100% cells with intermediate to strong stain). Positive (normal mouse brain) and negative (normal mucosa) controls were run in parallel for all experiments. The results were analyzed with Fisher’s exact test to determine the statistical significance of staining and tumor differentiation across experimental groups. A *p* value of <0.05 was considered significant. TUNEL (Roche), PCNA, Ki67 (Dakocytomation, #7240) and E-cadherin staining and analysis were performed as previously described ^[28, 33]^.

### Migration and invasion assays

Cells were serum-starved overnight and then 5×10^4^ cells were plated in cell culture insert wells (BD Falcon) or Matrigel-coated wells (BD Biocoat) under the described conditions. After 24 or 48 hours, the unmigrated cells on the upper chamber were removed and inserts were fixed and stained with Diff-Quik. For co-culture experiments, CAF cells were seeded into the lower chamber and tumor cells into the upper chamber of cell culture inserts, as above. After 24 or 48 hours, the unmigrated cells on the upper chamber were removed and inserts were fixed and stained with Diff-Quik. Images were captured (Cool-Pix digital camera, Nikon) and the degree of migration or invasion was determined by the average number of migrated cells in five 100× fields (ImagePro, Media Cybernetics). Experiments were performed in triplicate and repeated three times. Measurements were taken at indicated time-points and were quantified with ImagePro. Differences between groups were analyzed by utilizing either conventional Student’s *t* test or ANOVA followed by *post hoc* comparisons based upon modified Newman-Keuls-Student procedure, where appropriate. Results are reported as mean +/− SEM. A *p* value of < 0.05 was considered significant and all are two-tailed.

### Wound healing assay

Wound healing assay was performed using IBIDI (Troy, New York, USA) culture insert method in a six-well plate, as previously described ^[34]^. Cells (3×10^4^) were seeded in each of the reservoirs, grown overnight, and wound closure measurements were taken 24 hours following removal of the inserts. Experiments were performed in triplicate and repeated three times. Measurements were taken at indicated time-points and were quantified with ImagePro. Differences between groups were analyzed by utilizing either conventional Student’s *t* test or ANOVA followed by *post hoc* comparisons based upon Bonferroni’s Multiple Comparison Test, where appropriate. Results are reported as mean +/− SEM. A *p* value of < 0.05 was considered significant, and all are two-tailed.

### Gelatin zymography

Cells were seeded in 10-cm dishes and grown to 70% confluency as described previously ^[35]^. The next day, cells were washed twice with serum-free DMEM and cultured with serum-free medium containing BDNF (100ng/mL) for 24 Hrs. Supernatants were collected and centrifuged to remove cellular debris and the supernatant protein was concentrated with the Centricon 3 system.

### MMP-9 reporter gene Assays

The PGL3 construct containing the MMP-9-luciferase reporter gene was kindly provided by Dr. Gary Clayman (MD Anderson Cancer Center). HNSCC cell lines were transfected with the pGL3-MMP9-Luc construct in six-well plates, as described above. Cells were treated with CAF-derived conditioned media from CAF cells subjected to various treatment conditions as explained elsewhere. Cell lysates were prepared from each of the treatments and the luciferase activity was measured using Promega dual luciferase assay kit as per the instructions of the manufacturer (Promega Corporation, Madison, WI). All of the reporter assays were done in triplicate and repeated three times.

### Soft agar assays

Six-well culture plates were coated with 1 ml bottom agar mixture (DMEM with 20% FBS, 0.6% agar) and allowed to solidify at room temperature. Cells (3X10^3^) were suspended in DMEM agar mixture (20% FBS, 0.3% agar) and plated over the bottom layer of the 0.6% agar-medium mixture. After 12 days, plates were stained with 0.005% crystal violet and colonies larger than 100uM were counted.

### Experimental animals and orthotopic implantation of tumor cells

Male athymic nude mice (NCI-nu), aged 8–12 weeks, were purchased from the Animal Production Area of the National Cancer Institute-Frederick Cancer Research and Development Center (Frederick, MD, USA). The mice were used in accordance with Animal Care and Use Guidelines of The University of Texas M. D. Anderson Cancer Center under a protocol approved by the Institutional Animal Care and Use Committee. For establishment of HNSCC tumors, cells were injected into the tongue of athymic nude mice with 2.5×10^4^ cells, as described previously ^[30]^ or as a combination of HNSCC: CAF in a ratio of 1:1 or 1:5. For mechanistic studies mice were injected with OSC19-luciferase (Luc) (2.5×10^4^), combination of OSC19-Luc and CAF, combination of OSC19-Luc and CAF with NT shRNA or as a combination of OSC19-Luc and CAF with BDNF shRNA in a ratio of 1:1 or 1:5 HNSCC:CAF.

Tumor volume was measured weekly, and the differences between tumor volumes were evaluated by the non-parametric Mann–Whitney test. Results are reported as mean +/− SEM. A *p* value of < 0.05 was considered significant. Survival was calculated by the method of Kaplan and Meier. For the experimental model of metastasis, 1×10^5^ cells were injected into the tail vein of mice. At the end of all experiments, mice were sacrificed and tissues were collected for H&E staining and immunohistochemistry (IHC) analysis. TUNEL and Ki67 IHC staining and analysis were performed as previously described ^[30]^.

### Mouse imaging

Mice were imaged weekly with the IVIS Imaging System (*Xenogen,* Alameda, CA) after intraperitoneal administration of D-luciferin (*Xenogen, Alameda, CA)* ^[28]^. Results from the *in vivo* Luciferase assays were evaluated by the non-parametric Mann-Whitney test. Results are reported as mean +/− SEM. A *p* value of < 0.05 was considered significant and are two-tailed.

## Results

### Molecular characterization of CAFs

**Figure 1:**
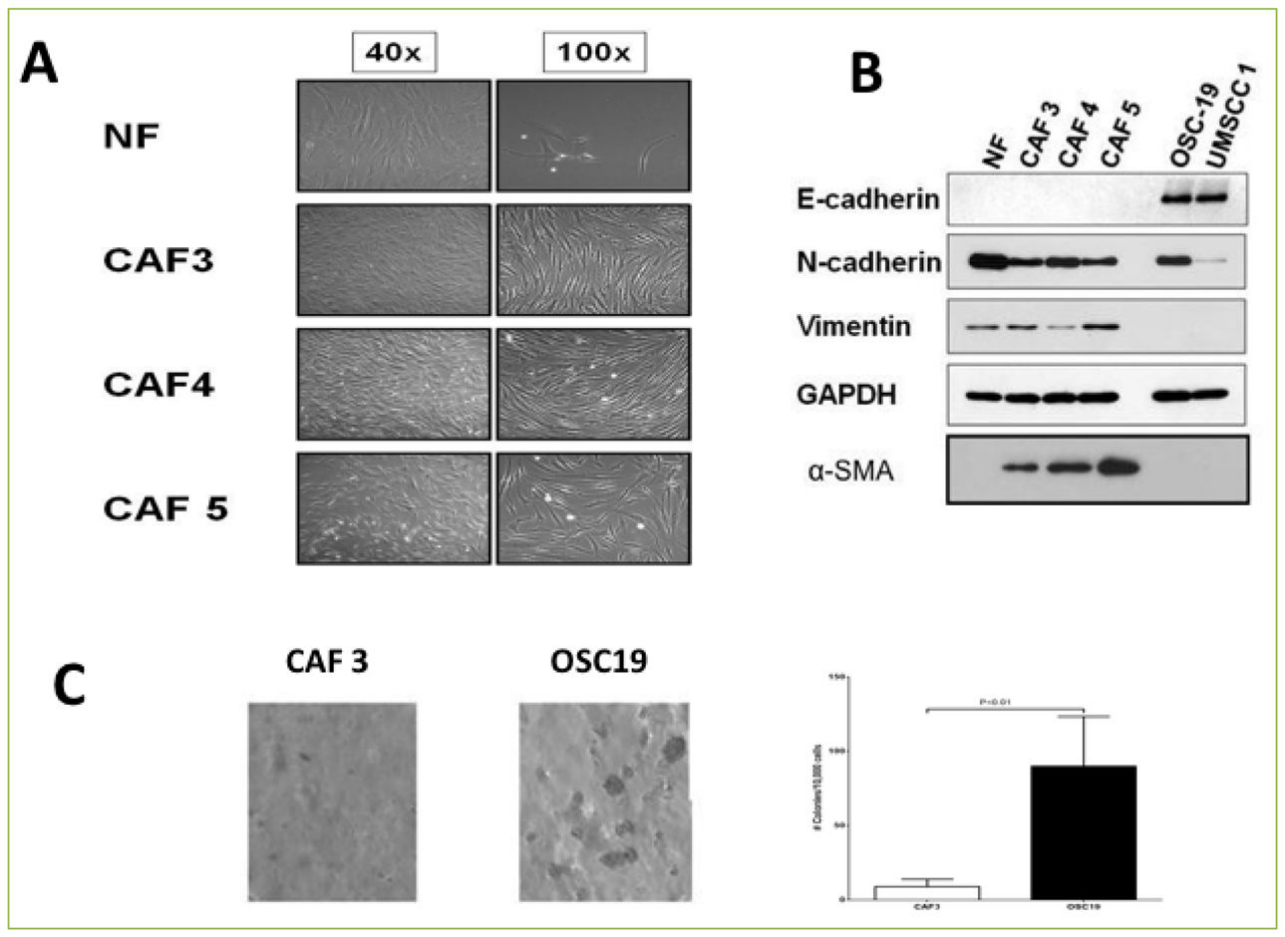
Cancer Associated Fibroblasts (CAFs) show morphological and biochemical characteristics of mesenchymal cells **A**. Normal fibroblasts and CAFs were allowed to grow in 10% DMEM media. CAFs show distinct morphology from normal fibroblasts. **B**. Western blot analysis of CAFs and epithelial cells showing mesenchymal markers in CAFs. **C**. Soft agar assay was used to detect the anchorage independent growth of CAFs and HNSCC cell lines. CAFs and HNSCC cells were suspended in 0.3% of top agar and added to a six-well plate containing a 0.6% solidified agar and incubated for 10 days to investigate anchorage independent colony formation. The gels were then stained with crystal violet stains and colonies lager than 100um were counted under a steriomicroscope. Data represent means ± S.E.M. Significance was determined by Student t-test.

We first characterized CAFs extracted from surgically-resected head and neck squamous cell carcinomas and compared their phenotypic properties to normal human fibroblasts (NF). Light microscopic analysis of three CAF cell lines (CAF-3, CAF-4, CAF-5) revealed cellular morphologies that were consistent with mesenchymal stromal cells (Figure 1A). The molecular profile of these cell lines was next compared to those from human HNSCC tumor cell lines and NF cell lines. Western blotting demonstrated an expression profile that was consistent with stromal fibroblasts, with minimal evidence of an epithelial derivation (Figure 1B). Soft agar experiments were performed to determine the tumorigenic potential of the derived cell lines, and only small cellular colonies developed, without evidence for focal spheroid formation, when compared to cancer cell lines (Figure 1C). CAF cells were next injected into mice to determine whether these cell lines were tumor-derived sub-populations of mesenchymal-like carcinoma cells, and none of the injected mice developed subcutaneous tumors (data not shown). These findings confirmed that the CAF cell lines were phenotypically consistent with fibroblasts and are non-tumorigenic in both *in vitro* and *in vivo* experimental conditions.

### CAFS induce potent HNSCC migration, invasion and therapy resistance

**Figure 2:**
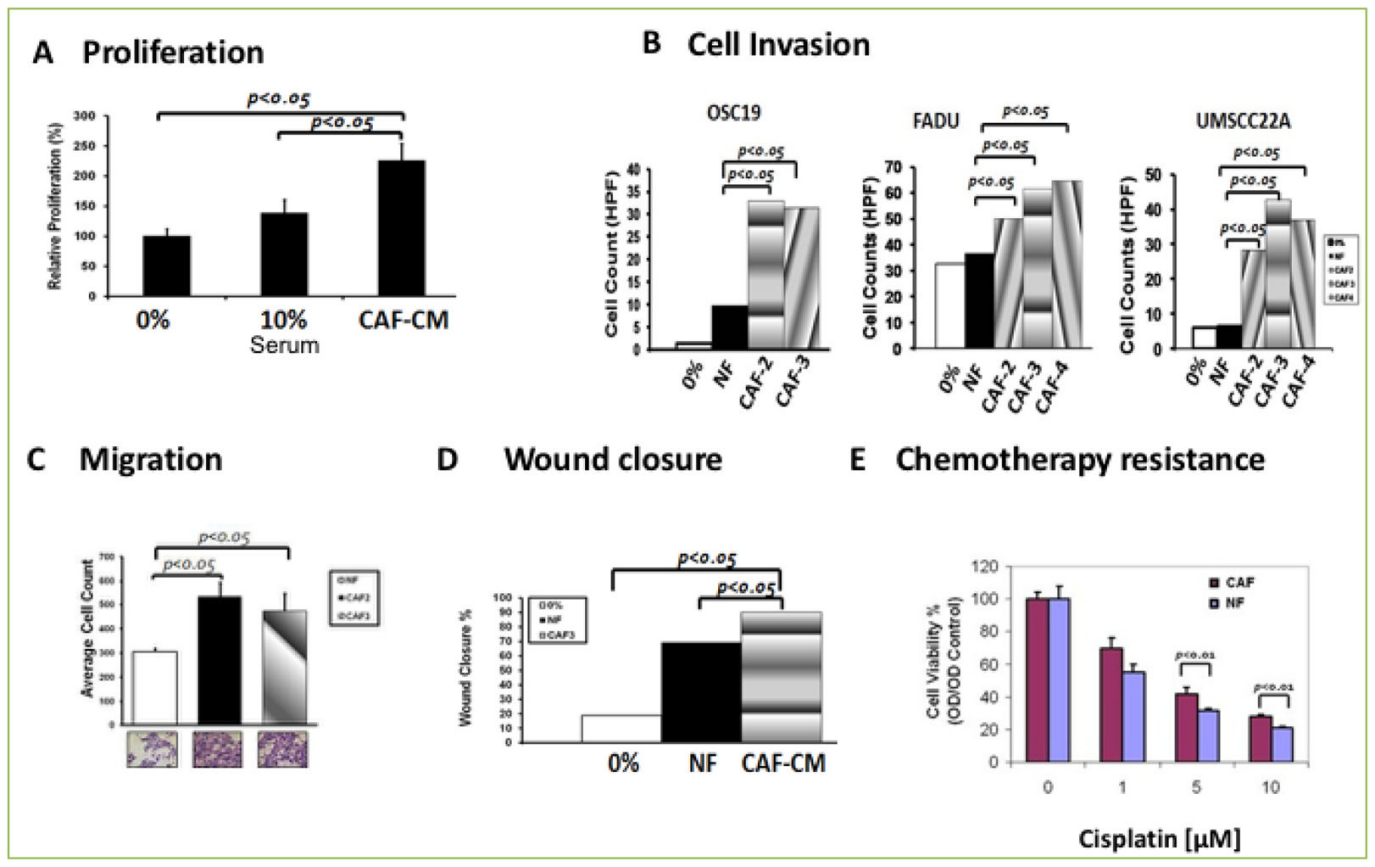
CAF potentiates HNSCC progression including Cell proliferation, migration, invasion, and chemotherapy resistance **A**. MTT assay showing a significant (p<0.05) induction of proliferation by CAF conditioned medium in HNSCC cell lines (P<0.05). **B**. Transwell co-culture of HNSCC cell lines with CAFs showed a significant increase in cell invasion of three HNSCC cell lines, OSC19, FADU, and UMSCC22A when compared to normal fibroblasts (P<0.05). **C**. HNSCC Cells line were co-cultured with/without CAF and migrating cells were counted. CAF induced significant migration (P<0.05). **D**. HNSCC cell line was grown to confluency in six well plates and wound scratch was made across the center of the wells. Cells were allowed to heal in the presence of conditioned medium from either of normal fibroblast (NF) or CAF. The distance between the two edges was measured at 0 time and 24 Hrs. Conditioned media from CAFs induced wound closure at a significantly faster rate (P<0.05) than cells incubated with CM from NF. **E**. HNSCC cell lines were incubated in the presence or absence of CM from NF or CAF in an increasing concentration of cisplatin. HNSCC cells incubated with CM from CAFs were more resistant than those incubated with CM from NF at higher concentration of cisplatin (P<0.05).

To determine whether the CAF-tumor synapse had unilateral functionality, we studied the impact of CAFs on the behavior of carcinoma cells in various cell-based assays. We first observed that secreted mediators from CAFs induced robust growth of HNSCC tumor cells in culture, when compared to standard concentrations of growth media (Figure 2A). We next determined whether tumor cells invaded in a chemotactic manner utilizing a model of tumor cell invasion in Matrigel Transwell assays. Consistently, CAF-CM potentiated basal cellular invasion in multiple cell lines. This finding was consistent across the OSC19, UMSCC22A and FADU cell lines (Figure 2B). We further extended these findings and observed that CAF-derived conditioned media significantly enhances HNSCC motility in Transwell experiments, when compared to normal fibroblasts (Figure 2C). Additionally, conditioned media derived from CAF cells potently activated cellular migration of HNSCC tumor cells in wound scratch assays, when compared to normal fibroblasts or standard media (Figure 2D). Finally, to determine whether CAFs induce relative resistance of HNSCC cells to standard cytotoxic therapy, tumor cells were exposed to increasing doses of the chemotherapeutic agent cisplatin, a standard cytotoxic for SCCA, in the presence of CAF serum. Uniformly, the addition of CAF conditioned media (CAF-CM), compared to normal fibroblast conditioned media (NF-CM), suppressed the anti-proliferative properties of this drug, suggesting that the clinical response to traditional systemic therapy may be abrogated by a robust CAF infiltrate (Figure 2E). Collectively, these experiments suggested that CAFs derived from HNSCC tumors have unique biological properties that are distinct from normal fibroblasts and contribute to the *in vitro* aggressive behavior of HNSCC.

### CAFs increase metastatic efficiency in HNSCC

**Figure 3:**
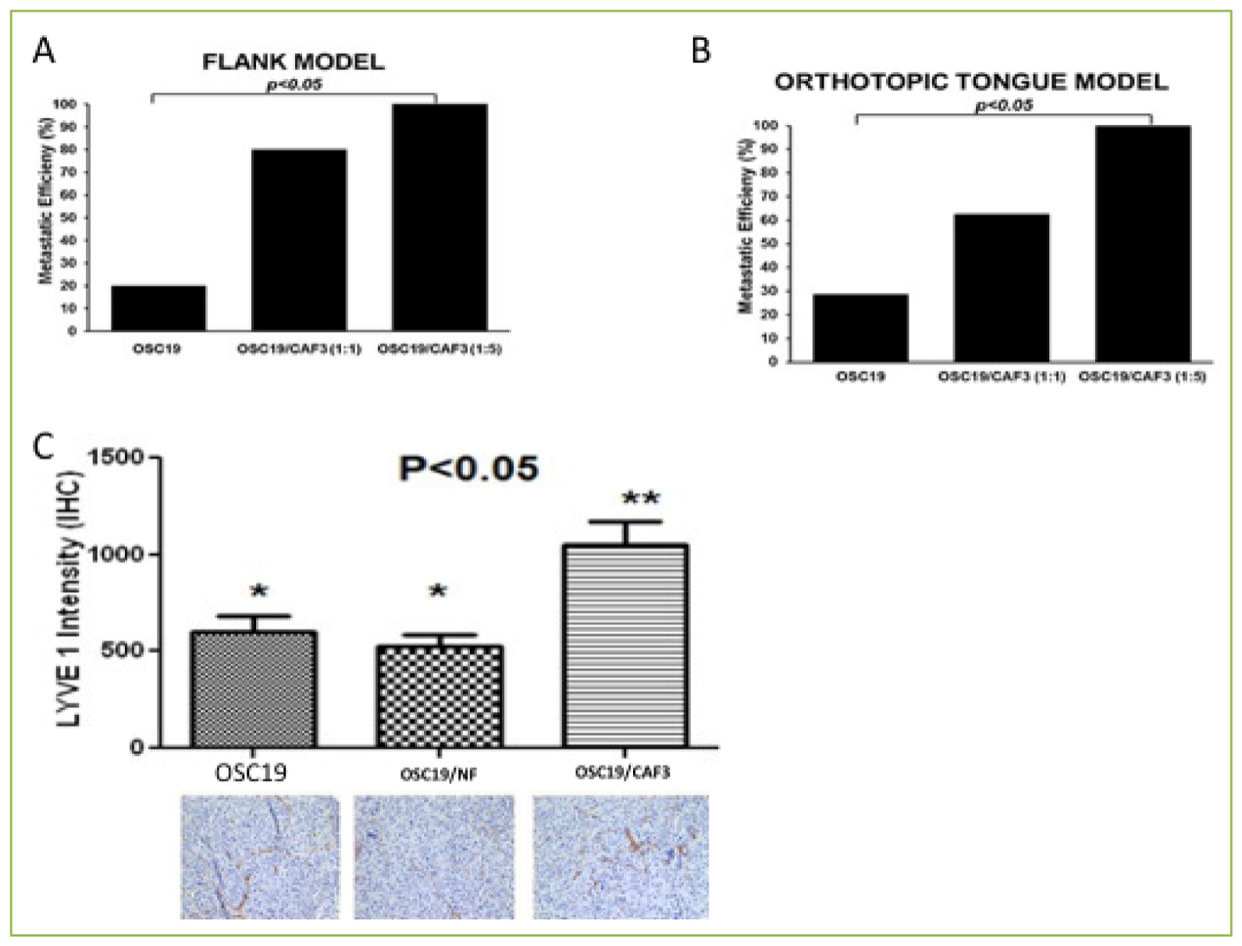
CAFs increase metastatic efficiency of HNSCC in mouse xenograft models **A–B**. Mice were injected with a suspension of OSC19, OSC19 and CAF (1:1) or OSC19 and CAF (1:5) in matrigel on the flank **(A)** or on the tongue **(B)** and maintained until the tumor growth reached 15mm as recommended by the institutional policy. Mice were sacrificed and adjoining lymph nodes and lungs were excised and processed for histological examination. Results indicated CAFs promoted metastatic efficiency (p<0.05) both in the flank model and the tongue model. **(C)** Immunohistochemical staining for LYVE1, a lymphangiogenic marker, was performed to measure the role of BDNF in potentiation of lymphangiogenesis in mice injected with OSC19, OSC19 and CAF transfected with vector or OSC19 and CAF transfected with BDNF construct. Overexpression of BDNF in CAFs significantly increased lymphangiogenic network as measured by LYVE1 intensity, p<0.05.

To evaluate the *in vivo* impact of CAF pathophysiology in the tumor microenvironment, we next extended these observations to animal models of lymphatic metastasis. Mice were injected with tumor cells alone, or were co-injected with CAF cells at various concentrations, utilizing the flank and orthotopic tongue models. In the flank model, the incidence of metastasis to regional nodes was enhanced when tumor cells were co-injected with CAFs. Utilizing either a dilution of 1:1 or 3:1, co-injection with CAF cells was associated with a statistically significant increased incidence of lymphatic metastasis (Figure 3A). Moreover, CAF cells alone were non-tumorigenic, and the CAFs induced a 2-fold increase in the metastatic efficiency of tumor cells when they were injected at a 1:5 ratio (tumor cells:CAFs; Supplementary Figure 1). In the orthotopic model of HNSCC, tumors were established in all tested groups, but the incidence of lymphovascular metastasis was enhanced in the CAF-injected groups (Figure 3B), and immunohistochemical staining for LYVE-1 confirmed the lymphatic micro-localization of metastatic deposits in the cervical soft tissues and in the peri-vascular spaces (Figure 3C). In addition, an analysis of intra-tumoral LYVE-1 revealed a significant enhancement of lymphatic endothelial infiltration. The results of these experiments suggested that CAFs play a crucial role in multi-stage tumor progression and revealed a novel role for CAFs in mediating metastasis through lymphovascular engagement in HNSCC.

### The CAF-tumor synapse is mediated by BDNF-TrkB signaling

**Figure 4:**
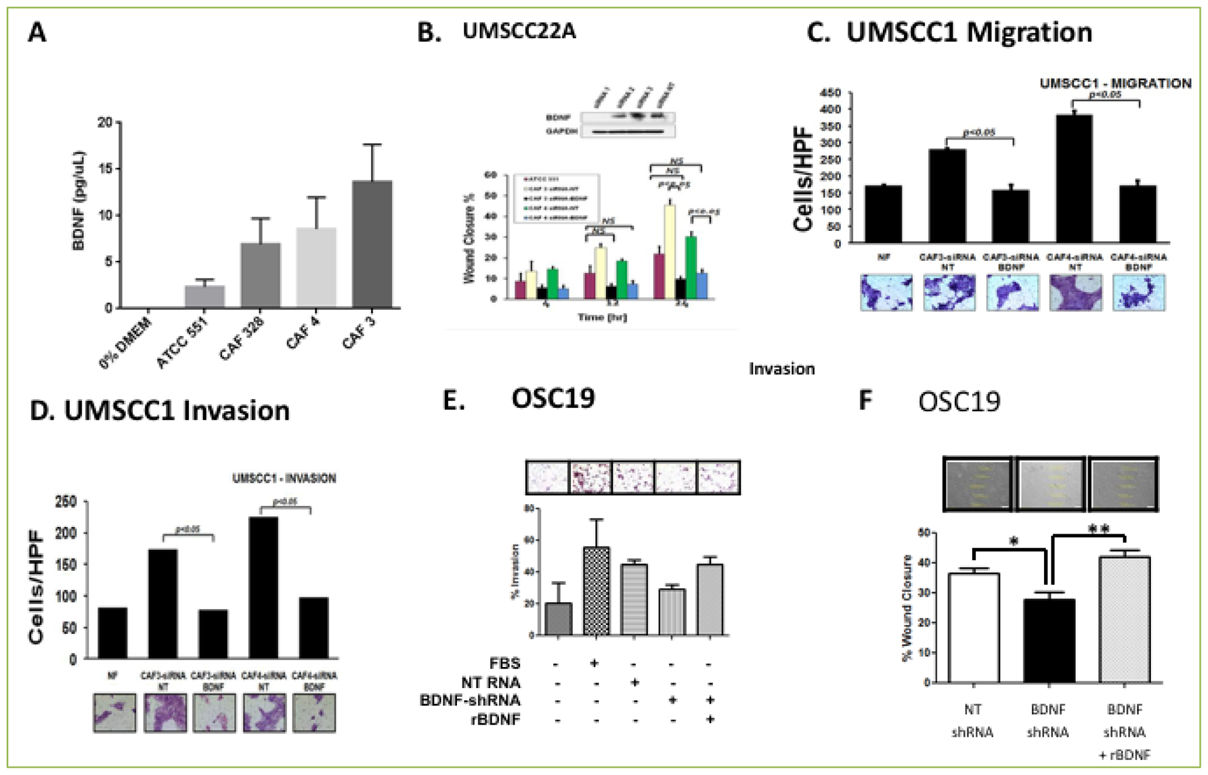
CAFs promote tumor cell migration, invasion and progression by activating the TRK-B-BDNF signaling axis **A**. ELISA was performed on supernatants derived from normal fibroblasts or CAFS for expression of BDNF and showed that there was a significantly higher level of BDNF secretion in CAFs (p<0.05). **B** HNCC cell line was grown to confluency and scratched for generation of wound. Wound healing was measured at 4, 12 and 24 Hrs in the presence of either NF or CAFs treated with non-targeting (NT) siRNA or BDNF siRNA. Wound healing was significantly inhibited in the presence of BDNF siRNA (P<0.05). **C–D**. Genetic suppression of BDNF in CAFs by using BDNF siRNA significantly reduces cellular migration (**C**) and Cellular invasion (**D**) in matrigel invasion assay (p<0.05). **E**. Matrigel invasion assay was performed on HNSCC lines incubated in medium derived from CAFs transfected with vector containing NT shRNA, BDNF shRNA or BDNF shRNA and recombinant BDNF. Addition of exogenous BDNF restored invasion of HNSCC cell line. **F**. HNSCC cells were grown to confluency in six well plates. Wound was generated by pipet tips along the diameter of the well. Reconstitution of exogenous BDNF (100nM) in the media restored wound healing in HNSCC cell line incubated with CM derived from CAFs transfected with NT shRNA or BDNF shRNA, p<0.05.

Our prior studies suggested that upregulation of TrkB signaling potentiated biological aggressiveness in HNSCC and was a putative target for this disease^[35]^. While some studies suggested that tumor-derived BDNF functioned as an autrocrine and paracrine ligand, this was not evident in HNSCC. We therefore hypothesized that the CAF-induced alterations in HNSCC behavior was due, in part, to CAF-secreted BDNF in the tumor microenvironment. To test this hypothesis, we first profiled the expression of various cytokines in multiple newly-established CAF cell lines resected from human tumors with both Western blotting and ELISA, and significant secretion of BDNF was noted in nearly all tested cell lines, compared to NF (Figure 4A). We also analyzed the expression and activation of receptor tyrosine kinases (RTK) in CAF cell lines and, in comparison to NF, strong TrkB receptor function was identified, in addition to c-MET activity (data not shown). These findings suggested that BDNF signaling operates in the tumor microenvironment through proactive nature of the cancer-associated fibroblastic compartment, in comparison to the relatively quiescent normal fibroblasts, in the tumor microenvironment.

### Abrogation of BDNF targets CAF-directed HNSCC behavior

To directly test the hypothesis that an active CAF-derived BDNF axis specifically regulates the oncogenic milieu, we pharmacologically and genetically inhibited BDNF signaling and interrogated the biological response of the tumor compartment *in vitro*. A small molecular inhibitor of TrkB was added to CAF-CM and then exposed to HNSCC cells in culture ^[36]^. In both the OSC19 and UMSCC22A cell lines, wound closure was significantly inhibited in cells treated with the TrkB inhibitor, compared to CAF-CM alone, after 24 hours (Supplementary Figure 2). This effect was dose dependent as well, and provided further evidence of an active neurotrophin system in the CAF-tumor dynamic.

To further distinguish the contribution of BDNF in the microenvironment, we selectively inhibited BDNF expression with siRNA constructs targeting BDNF. Conditioned media from CAFs treated with a non-targeting construct (siRNA-NT) or siRNA targeting BDNF (siRNA-BDNF) was added to HNSCC cells growing in culture. The wound scratch assay revealed significant impairment of HNSCC migration under conditions of BDNF knockdown in the CAF-CM, suggesting that potentiation of cellular motility is due in part to paracrine communication between tumor and stromal cells through BDNF-TrkB (Figure 4B). We next explored whether chemotactic migration and invasion were also directly affected by CAF-derived BDNF. Tumor cells exposed to BDNF-depleted CAF-CM (siRNA-BDNF) had a 50–75% reduction of migration (Figure 4C, Supplementary figure 3) and invasion (Figure 4D), compared to a non-targeting construct. Additionally, this degree of impairment closely approximated the effects of stimulation with CM-NF, suggesting that upregulation of BDNF expression in CAFs was in part responsible for the hyperdynamic behavior of tumor cells.

To determine whether the effects of CAFs on HNSCC cells in the tumor microenvironment were directly attributable to CAF-secreted BDNF, we performed BDNF rescue experiments under *in vitro* conditions. We first stably inhibited the expression of BDNF in the CAF compartment under in vitro conditions with sh-RNA constructs. Cancer cells were exposed to medium from either BDNF-depleted CAFs (BDNF-shRNA) or non-targeted CAFs (NT-shRNA). In Matrigel invasion experiments, tumor cells exposed to BDNF-depleted CAF media has significantly decreased cellular invasion compared to media from non-targeted CAFs. This effect, however, was reversed when exogenous recombinant BDNF was added to the experimental conditions (Figure 4E). In complementary experiments, the inhibitory effects of BDNF depletion in CAF cells was completely reversed with exogenous ligand in a wound scratch assay (Figure 4F). These two rescue experiments further established the direct regulation of cancer cell migration in the tumor microenvironment through a BDNF-directed CAF-tumor interface.

### MMP-9 in the tumor compartment is activated by CAF-secreted BDNF

**Figure 5:**
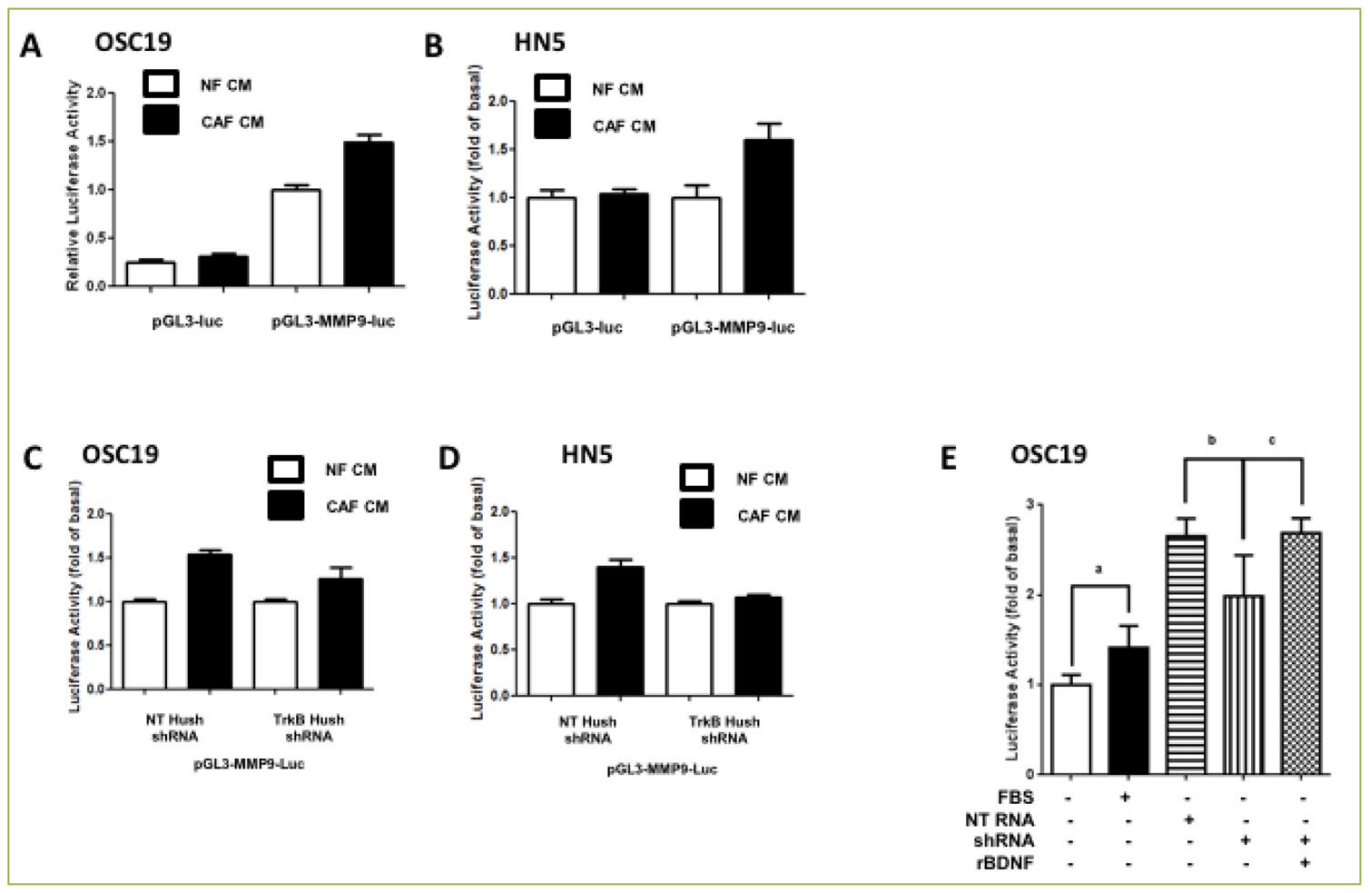
BDNF/TrkB system mediates CAF-induced MMP9 expression in HNSCC cell lines **A–B**. HNSCC cell lines were transfected with either empty vector (PGL3-Luc) or vector containing MMP9 promoter region (PGL3/MMP9-Luc) and incubated in conditioned media derived from normal fibroblast (NF-CM) or CAF (CAF-CM) for 24 Hrs. Cell lysates were collected and luciferase activity was measured for OSC19 **(A)** or HN5 **(B)**. While conditioned media derived from NF failed to induce MMP9 activity, CAF-CM derived from CAFs transfected with MMP9 luciferase promoter region induced significantly higher level of luciferase activity, p<0.05. **C–D**. HNSCC cell line transfected with vector containing NT shRNA or TRK-B shRNA was doubly transfected with vector containing MMP9-Luc (pGL3-MMP9-Luc) and grown for 24 Hrs in NF-CM or CAF-CM. Inhibition of TRK-B expression in HNSCC cell lines by using TRKB shRNA attenuated MMP9 activity to similar level as conditioned media derived from a normal fibroblast. **E**. Exogenous application of recombinant BDNF in OSC19 restores MMP9 activity.

To determine more directly the role of BDNF-TrkB in activating the pro-migratory and pro-invasive phenotype induced by CAFs, we explored the downstream pathways active in this phenotype. In SCCA, MMP-9 has been directly linked to the progression of tumors ^[30]^, and CAFs play in role in modulating the activation of this axis ^[37]^. Our prior studies demonstrated that exogenous BDNF upregulated MMP9 expression and activity *in vitro*. (35) To test the hypothesis that MMP-9 expression in the tumor compartment can be modulated by BDNF sourced from the CAF compartment, we explored the impact of alterations in the fibroblast secretome on this pathway. After transfection with an MMP-9 promoter construct linked to a luciferase reporter gene, HNSCC cell lines were exposed to conditioned media from either NF or CAF cells. When analyzed by luciferase activity, HNSCC cells exposed to CAF-CM had substantial upregulation of MMP-9 promoter activity compared to NF-exposed cell lines (Figure 5A & B). We next tested the hypothesis that modulation of BDNF in the CAF compartment would profoundly impact MMP-9 activity in the tumor compartment. HNSCC cell lines were exposed to conditioned media from either NF or CAF cells that had been genetically modified to suppress BDNF expression. When analyzed by luciferase activity, HNSCC cells exposed to BDNF-depleted CAF-CM had depressed signal intensity compared to the CM from null-transfected CAF cells (Figures 5C & D). Rescue experiments were performed, where BDNF was re-introduced in the BDNF-depleted CM, which re-establishment of the basal levels of luminescent activity, comparable to the control condition (Figure 5E). These results demonstrated that CAFs activate an intercellular program of enhance ECM degradation in the tumor compartment, mediated in part through a BDNF-TrkB axis.

### Suppression of CAF signaling limits SCCA tumor growth

**Figure 6:**
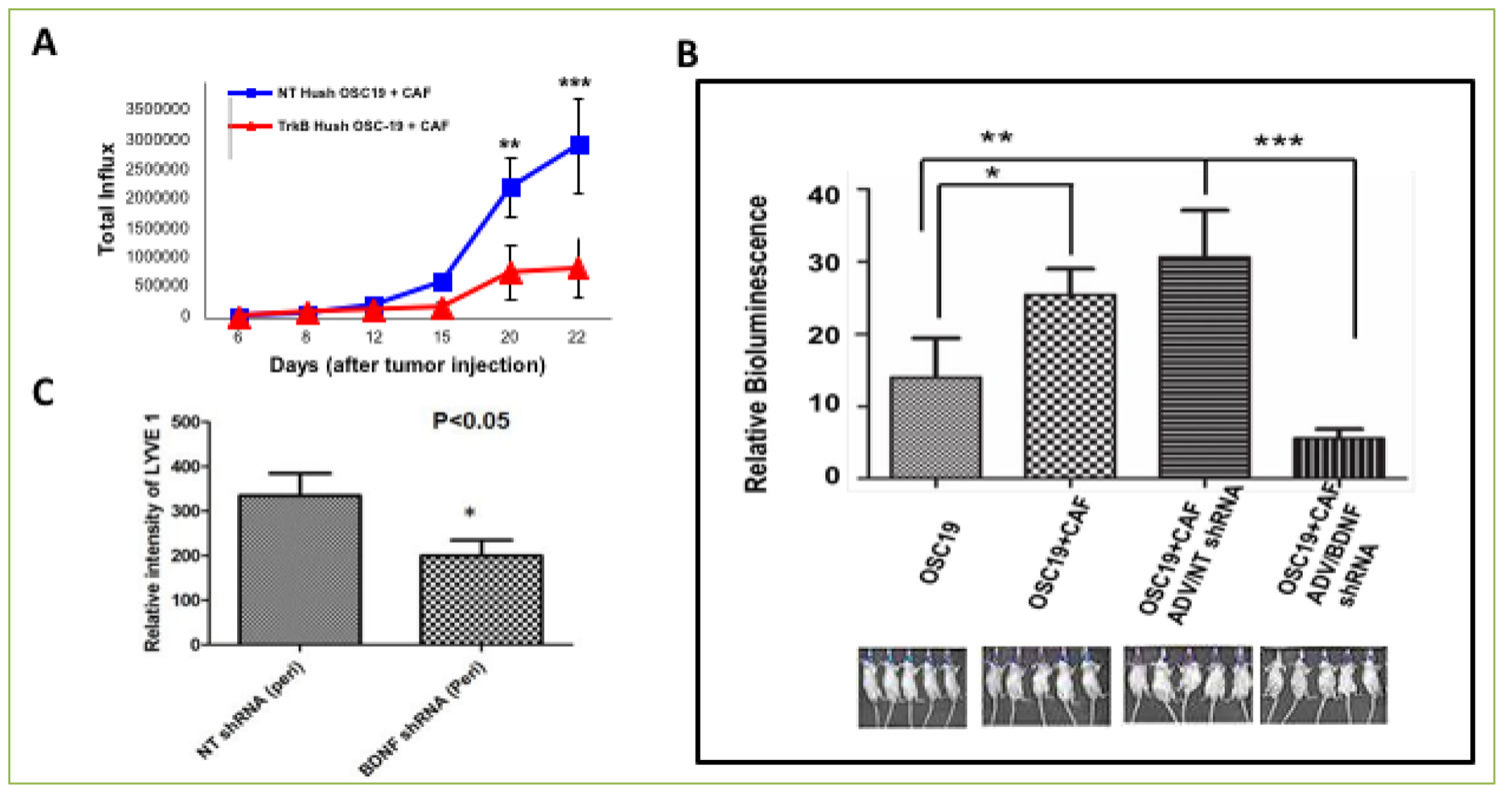
CAFs promote tumor growth in mouse orthotopic models through TRK-B-BDNF signaling axis **A.** Mice were co-injected in the tongue with OSC19-Luc transfected with a combination of NT shRNA or TRK-B shRNA and CAF to investigate the role of TRKB derived from HNSCC. Inhibition of TRKB in HNSCC significantly reduced tumor growth even in the presence of CAF. **B**. Mice were injected with OSC19 harboring luciferase (Luc) (2.5×10^4^) construct alone or OSC19 harboring luciferase construct with CAF, or with combination of OSC19-Luc and CAF with NT shRNA or a combination of OSC19-Luc and CAF with BDNF shRNA. Tumor growth was followed by measuring relative bioluminescence. A significantly higher *in vivo* bioluminescence intensity was correlated with co-injection of a combination of OSC19-Luc and CAF, p<0.05. However, tumor growth was significantly reduced in mice injected with a combination of OSC19-Luc and CAF in which expression of BDNF is inhibited by shRNA, p<0.001. **C**. Immunohistochemical staining for LYVE1 was performed on tissue sections derived from xenografts of mice co-injected with OSC19 luciferase and CAF harboring non-targeting HuSH or with OSC19-Luc and CAF harboring anti-BDNF Hush. Lymphangiogenic network was measured in the peritumoral region. Results showed that reduction of tumor growth in mice injected with a combination of OSC19-Luc and CAF/BDNF shRNA was associated with a significant decrease in the expression of LYVE, p<0.05.

To extend our findings in a relevant animal model, we next evaluated the impact of altered BDNF function in the tumor microenvironment. HNSCC cells harboring either a TrkB-inhibitory shRNA construct, or a non-targeting construct, were co-injected with CAF cells into the tongues of nude mice. Serial measurements of cancer development with *in vivo* bioluminescence revealed abrogated growth in mice injected with shRNA-TrkB and CAF cells, compared to mice injected with shRNA-NT and CAF cells (Figure 6A). We further analyzed the impact of CAFs on the development of metastasis, and found that when BDNF signaling was modulated in the tumor microenvironment, regional metastasis and metastatic efficiency were impaired (Figure 6B). While these studies do not exclude the possibility that the limited tumor growth in the shRNA-TrkB cells was due in part to the partial loss of TrkB function in the tumor cells, the magnitude of the impact on lymphatic metastasis cannot be explained entirely by the loss of TrkB. Thus, these results suggest that CAF stimulatory influence cannot overcome these effects *in vivo*. Moreover, the infiltration of tumoral lymphatics was significantly downmodulated (Figure 6C), suggesting that the absence of a functional BDNF-TrkB axis in the tumor milieu has a negative effect on lymphovascular recruitment and on the progression to lymph node metastasis in this orthotopic model.

To definitively demonstrate that CAF-derived BDNF impacted tumor growth and lymphatic metastasis, we selectively down-modulated BDNF expression in the CAF compartment in an animal model of head and neck cancer. After stable transfection with shRNA constructs targeting BDNF (shRNA-BDNF) or a non-targeting sequence (shRNA-NT), CAF cells were co-injected at a 5:1 ratio with HNSCC cell lines in the orthotopic tongue model. Co-injection of CAFs harboring an shRNA-NT construct with HNSCC cells demonstrated enhanced tumor growth, compared to co-injection of CAFs harboring an shRNA-BDNF construct. The suppression of BDNF in the CAF compartment led to decreased tumor growth, suggesting that CAF-derived BDNF is a critical mediator of in vivo tumor growth. These findings also support the notion that BDNF is required for tumor growth, but is not a mediator of tumor engraftment.

### BDNF expression is associated with metastasis in HNSCC

As our experimental evidence revealed an important regulatory role for BDNF in the tumor microenvironment, we sought to determine the clinical relevance of BDNF as a marker of metastasis in HNSCC. Utilizing a tissue array of clinically-annotated uniformly treated HNSCC patients, we assayed human HNSCC tumors for the expression of BDNF and correlated these findings to clinical outcomes. In a set of 71 HNSCC tumor patients, BDNF expression was increased (≥ *+1* score) in 17% of tumors that were analyzed. On univariate analysis, the development of metastasis was significantly associated with BDNF expression, although an effect on survival was not demonstrable. We further identified that expression of LYVE-1 was significantly associated with BDNF expression in human HNSCC, suggesting that BDNF upregulation may induce LYVE-1-mediated lymphatic network establishment in this disease (Supplementary figure 4). These results suggested that in HNSCC, the neurotrophin axis was an adverse prognostic factor for the development of metastasis.

## Discussion

In this study, we identify novel mechanisms for fibroblast-induced metastasis in human cancers, primarily via a paracrine neurotrophin axis in the tumor microenvironment. These conclusions are based upon the following critical findings: (1) human-derived cancer associated fibroblasts potentiate *in vitro* invasion and *in vivo* metastasis in models of squamous cell cancer (2) phenotypic behavioral changes induced by CAFs are coordinated by BDNF-mediated TrkB signaling axis cascade that incites a pro-invasive expression profile (3) regional metastasis in mouse models of SCC is regulated in part by BDNF-TrkB signaling in the tumor microenvironment via CAFs (4) stromal-specific BDNF expression predicts outcomes for patients with SCC.

Neurotrophin and their receptors have been directly implicated in tumor progression and hematogenous metastasis. We previously demonstrated that TrkB activation induced EMT and invasion in animal models of OCSCC, and other studies indicate that TrkB overexpression directly mediates the metastatic phenotype in murine tail-vein and subcutaneous models of cancer progression ^[26, 27]^. We have also demonstrated the contribution of BDNF and TrkB to a chemo-resistant phenotype ^[38]^, which is associated with the potentiation of regional and distant metastases ^[39, 40]^. More recently, a provocative study of glioblastoma demonstrated that Trk receptors are targets for stromal-derived NTs the microenvironment ^[41]^. However, what has been lacking is an understanding of how CAFs contribute to the development of metastasis to the regional lymph nodes. In this study, we sought to directly link neurotrophin secretion from CAFs to the development of lymphatic metastasis.

With regional lymph node metastasis often being the most important prognostic marker for patients with epithelial cancers, several studies have implicated VEGF-C as a biomarker of adverse prognosis for multiple cancers ^[42–46]^. Other work has revealed a relationship between CAFs and lymphatic metastasis, but whether this remains a biological association is unclear, and a precise linkage mechanism remains elusive. Circumstantial evidence for this includes: (1) VEGF-C expression in CAFs is associated with increased nodal metastasis ^[45]^, (2) inhibition of CAF-secreted ECM components is associated with abrogated lymphangiogenesis ^[47]^ and (3) direct molecular targeting of CAFs inhibits lymphangiogenesis though an immunomodulatory mechanism. While these studies demonstrate the implicit importance of CAFs, the complex nature of human-tumor derived CAFs and their impact in murine tumor models remains poorly studied. Our data suggest that CAFs play a complex role in tumor progression that can be modulated by manipulation of the BDNF intercellular signaling system, mediated in part through alterations in pro-lymphangiogenic cascade.

There is evidence to suggest the presence of a BDNF signaling axis in the microenvironment of non-epithelial tumors, but the source of this ligand has been elusive ^[48]^. Moreover, validation in epithelial cancers and in pre-clinical models has not been established. The mechanisms by which CAFs mediate growth in vivo have also been studied in some models, and a link to angiogenesis has been proposed. While MMP2- and MMP9-deificent embryonic fibroblasts co-injected with SCCA cells demonstrated suppressed tumor growth in a mouse model of cancer, an enhancement of tumor establishment was noted, suggesting that CAF are sufficient, but not necessary, for tumor development ^[49]^. This finding has been confirmed in other systems, including pancreatic ^[50]^ and lung cancers. In other animal models, fibroblast-derived MMP-1 activates the PAR-1 receptor, resulting in tumor invasion and angiogenesis in breast cancer ^[51]^. However, a link between CAFs and lymphatic metastasis has not been established to date, although substantial evidence supports a role for CAFs in vascular angiogenesis. The goals of this study were to determine whether the biological impact of CAFs extended to lymphatic metastasis in relevant models of human cancer. In this realm, out findings support the novel notion that the multi-step metastatic process in HNSCC is directly regulated in part by a unique CAF-tumor interface.

In the CAF compartment, pre-clinical transgenic models have demonstrated that FAP regulates collagenase activity, and is important for angiogenesis and tumor growth ^[6]^. In a model of experimental metastasis, “priming” of tumor cells with fibroblast media potentiated engraftment of hematogenous metastases to lungs, a process mediated in part by TGF-β ^[7]^. In breast cancer, CAFs mediate pro- “estrogenic” effects through activation of the ERK pathway, suggesting a bi-directional interaction, although how this exists in mouse models has yet to be defined ^[8]^. Some of the most convincing in vivo data on a specific role for CAFs has been demonstrated in a *FAP*-deficient model of lung and colon cancer, with the demonstration that CAF-derived FAP directly modulates tumor growth and angiogenesis ^[6]^. One of the primary drivers of CAF-directed tumor progression appears to be an FGF-PGDFR intercellular circuit, which has been effectively targeted in a transgenic mouse model of cervical SCCA ^[9]^. Extending these findings to models of cutaneous SCCA, Erez *et al* identified that the step-wise progression from hyperplasia to cancer was controlled, in part, by an NF-κB inflammatory expression profile. Whether there is co-migration of CAFS with tumor cells to sites of metastasis remains unstudied, but an analysis of activated fibroblasts in rheumatoid arthritis revealed that pathologic fibroblasts are capable of “metastasis” to other joints, and similar phenotype in CAFs would not be impossible ^[52]^. In our study, we did not observe co-migration of CAFs to metastatic foci, and thus our findings suggest that the pro-metastatic impact of CAF occurs in the tumor micro-environment.

The mechanisms by which CAFs mediate tumor progression *in vivo* have been studied in some models, and a link to angiogenesis has been proposed ^[43]^. Mediators of CAF-mediated metastasis in animal models of non-squamous cancers have been observed, and include TGF-β ^[7]^, estrogen-ERK ^[8]^, and FAP ^[6]^. In selected squamous cancers, FGF-PGDFR intercellular circuitry ^[9]^ and an NF-κB inflammatory expression profile ^[10]^ can control the step-wise progression from hyperplasia to cancer. While MMP-deficient embryonic fibroblasts co-injected with HNSCC cells demonstrated suppressed tumor growth in a mouse model of cancer, an enhancement of tumor establishment was noted, suggesting that CAF are sufficient, but not necessary, for tumor development ^[49]^. This finding has been confirmed in other systems, including pancreatic ^[50]^, lung and breast cancers ^[51]^. In our study, we have explored the novel hypothesis that lymphatic metastasis may be mediated through a BDNF-TrkB intercellular signaling system. It is likely that parallel interactions and signaling redundancies exist, and thus further exploration of how the neurotrophin signaling is modulated by FGF and FAP warrant further investigation.

## Supplementary Material
