# Supplementary material for: Brain derived neutrophic factor (BDNF) coordinates lympho-vascular metastasis through a fibroblast-governed paracrine axis in the tumor microenvironment
Source: Cancer Cell Microenviron. Author manuscript; Available in PMC 2017 Sep 27. (PMC5617346; doi:10.14800/ccm.1566)
Supplement: Supplementary file 1 [file NIHMS894244-supplement-supplement_1.pdf]

Supplements

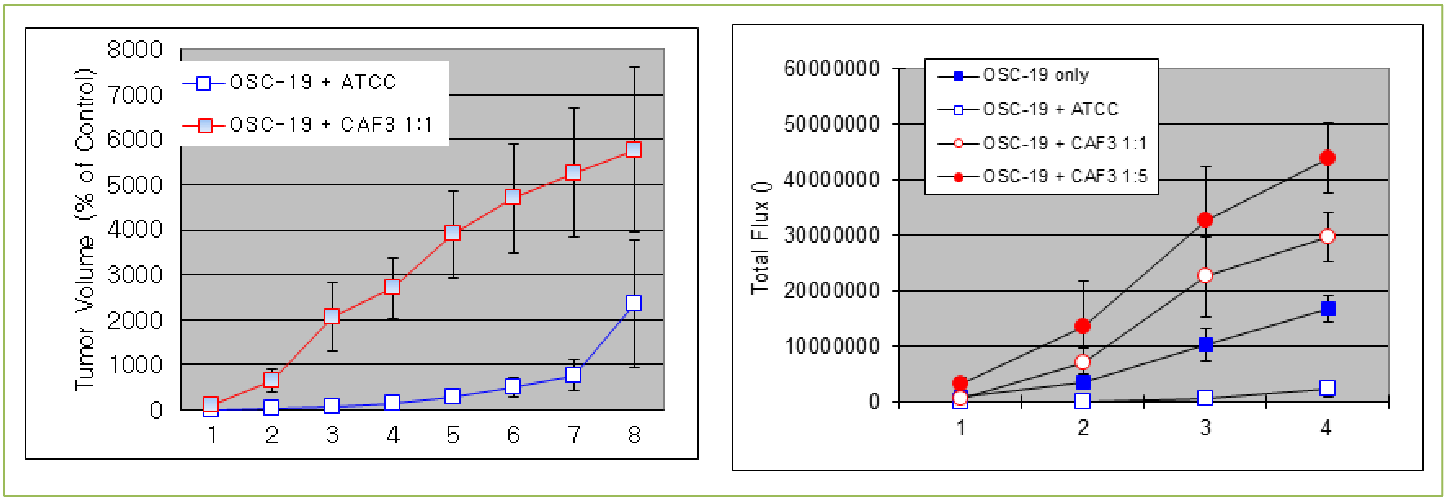

S1. *In vivo* fluorescence imaging of orthotopic mouse models implanted with either OSC19 and normal fibroblast or in combination with different ratios of CAFs. Fluorescence signals of the tumors were quantified by using IVIS200 software. Total flux was calculated for the groups.

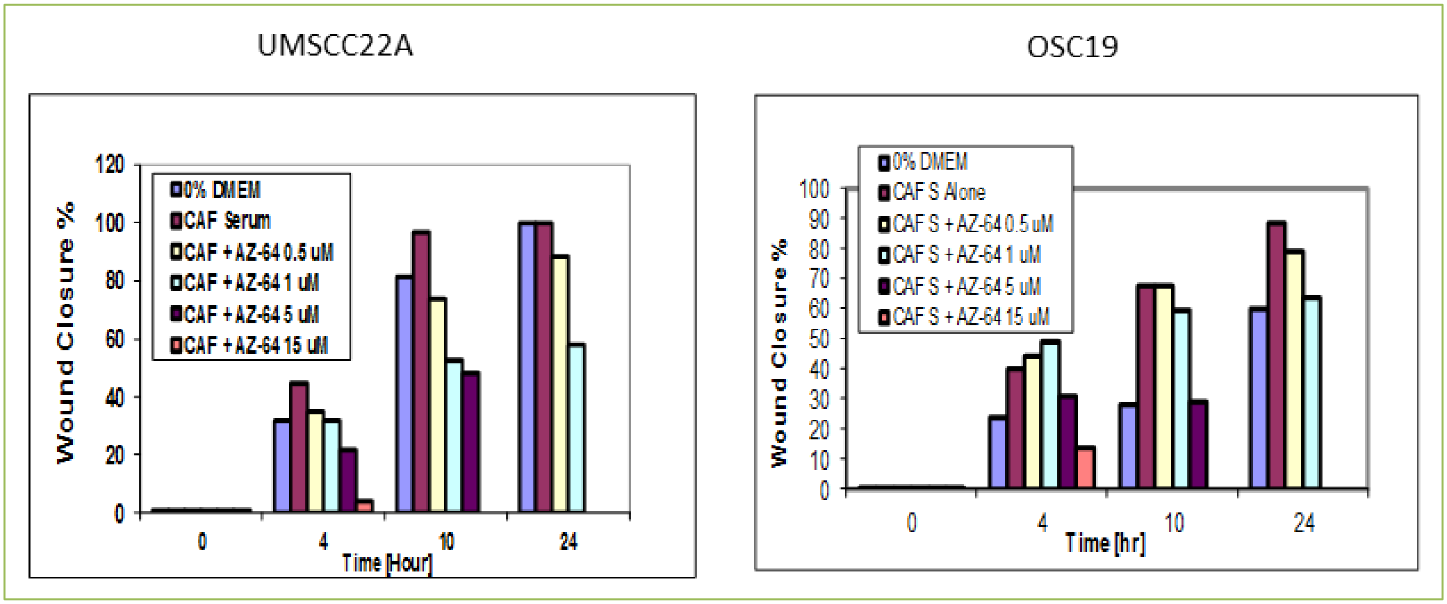

S2. HNSCC cell lines, OSC19 and UMSCC22A), were grown to confluency in six well plates and a wound scratch was made across the center of the well. Cells were allowed to heal in the presence of conditioned medium from CAFs treated with increasing concentration of AZ-64 (TRKB inhibitor). A distance was measured between the two edges at 0, 4, 10 and 24 Hrs. Conditioned media from untreated CAFs induced wound closure at a significantly faster rate than cells incubated with CM from CAFs treated with the TRKB inhibitor.

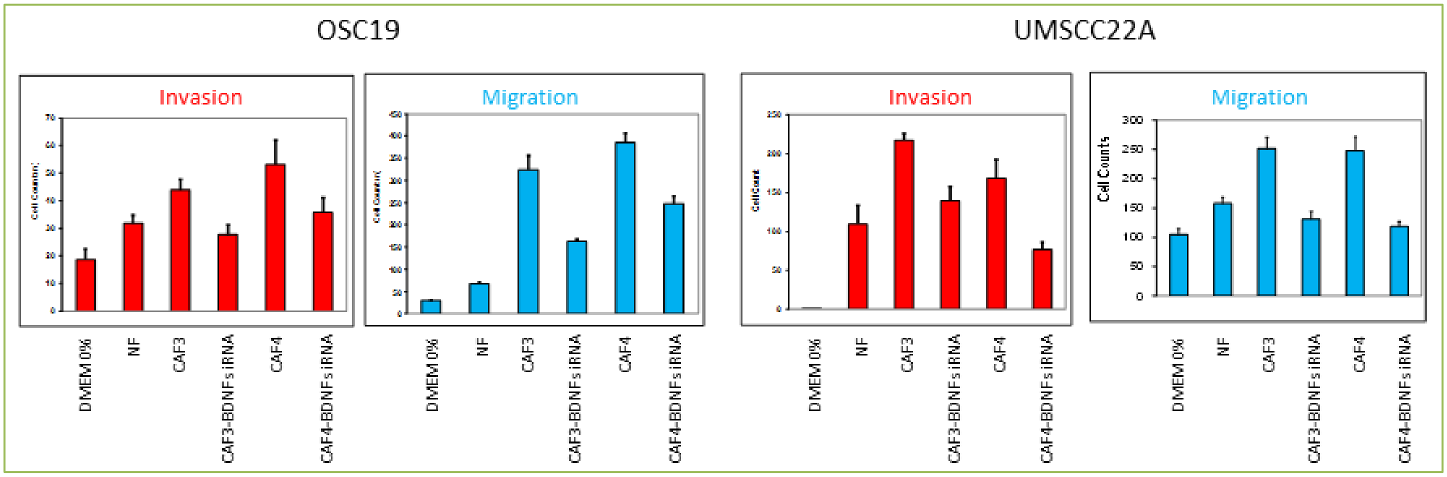

S3. Suppression of BDNF through siRNA in CAFS inhibits migration and invasion of HNSCC cell lines, OSC19 and UMSCC22A when cells are incubated with the conditioned media derived from normal CAF supernatant or from CAFs in which BDNF has been suppressed by treatment with BDNF siRNA.

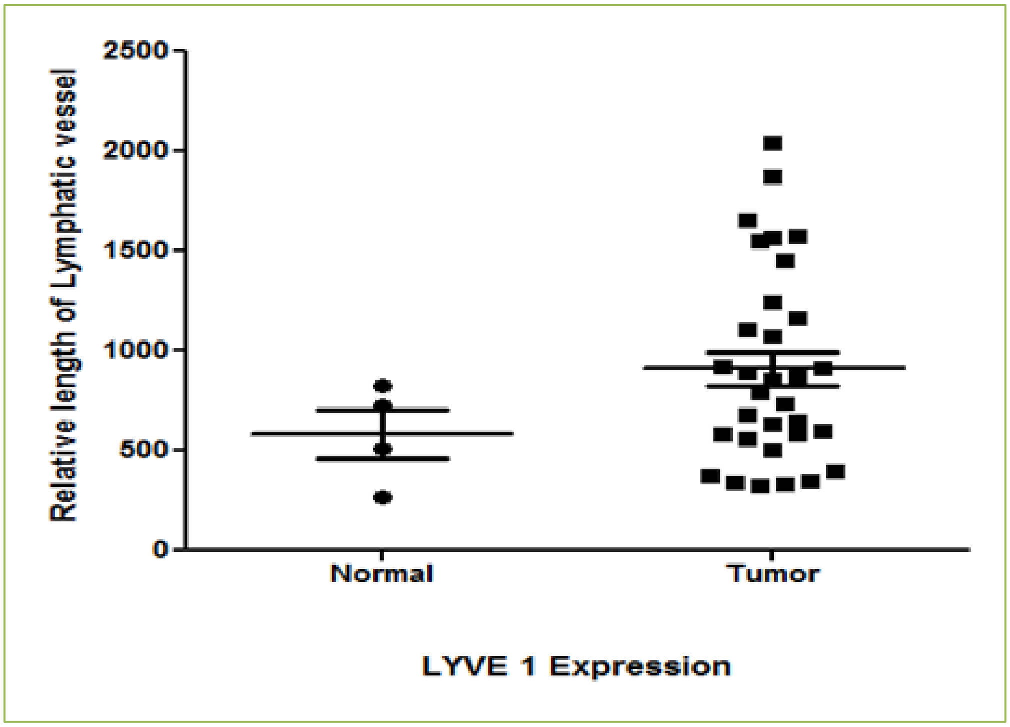

S4. Human squamous cell carcinoma tissue sections as well as matched normal tissue were stained with LYVE-1 antibody to measure lymphovascular extension.
